# Local-Scale Soil Heterogeneity Differentially Influenced Assimilative Branch Stoichiometry of Three Dominant Shrubs in a Central Asian Desert

**DOI:** 10.3390/plants14213363

**Published:** 2025-11-03

**Authors:** Cheng-Cheng Wang, Xue-Lian Zhang, Ye Tao, Ling Dai, Huan-Huan Meng, Xiao-Bing Zhou, Yuan-Ming Zhang

**Affiliations:** 1 College of Life Sciences, Tarim University, Alar 843300, China; wangchengcheng@163.com; 2 State Key Laboratory of Ecological Safety and Sustainable Development in Arid Lands, Xinjiang Institute of Ecology and Geography, Chinese Academy of Sciences, Urumqi 830011, China; zhangxuelian@163.com (X.-L.Z.); dailing_2025@163.com (L.D.); menghuanhuan1214@163.com (H.-H.M.); zhouxb@ms.xjb.ac.cn (X.-B.Z.); 3 Xinjiang Key Laboratory of Biodiversity Conservation and Application in Arid Lands, Xinjiang Institute of Ecology and Geography, Chinese Academy of Sciences, Urumqi 830011, China; 4 Xinjiang Key Laboratory of Special Species Conservation and Regulatory Biology, Key Laboratory of Special Environment Biodiversity Application and Regulation in Xinjiang, Key Laboratory of Plant Stress Biology in Arid Land, College of Life Sciences, Xinjiang Normal University, Urumqi 830054, China

**Keywords:** heterogeneous soil condition, desert shrub, assimilative branch, nutrient stoichiometry, influencing factor, interaction effect

## Abstract

Most traits of assimilative branches (ABs) present large spatial and interspecific differences; however, it is still unclear how small-scale soil heterogeneity influences nutrient traits in ABs under the same climatic conditions. The AB samples of *Ephedra przewalskii* (EP; small-sized), *Calligonum mongolicum* (CM; medium-sized), and *Haloxylon persicum* (HP; large-sized), as well as soil samples, were collected at three sites (north, middle, and south; within 65 km) in the southeastern Gurbantunggut Desert, China. The interspecific and inter-site differences in C:N:P:K stoichiometry and the relationships with soil properties were discussed. From north to south, soil nutrients and biocrust development improved, whereas coarse sand proportion decreased. Species and site markedly influenced ABs’ stoichiometry, with a significant interaction. At the species level, each stoichiometric trait differed among species. CM exhibited the lowest C:P and N:P, whereas HP had the highest N:P. At the site level, N:P and C:P of EP and CM increased from north to south, whereas HP changed unclearly. CM and HP had higher N–P scaling exponents, EP and CM exhibited a higher K allocation rate, resulting in the co-limitation of N and P for all species. The overall stoichiometric homeostasis ranked as follows: HP > CM > EP. The three shrubs were dispersed among each other in an ordination diagram based on nutrient metrics, with different distribution patterns. The nutrient traits in the ABs of EP and CM, rather than HP, were markedly correlated with most soil factors. Local-scale soil variation indeed influenced the nutrient strategies of desert shrubs; plant size might be another important factor.

## 1. Introduction

Carbon (C), nitrogen (N), phosphorus (P), and potassium (K) are indispensable elements for plant growth, development, reproduction, and other life activities [1]. C is the basis of the organic skeleton, and is also the substrate and energy source of various physiological and biochemical processes of plants; N and P are important components of protein and genetic material, respectively; and K is not only involved in regulating ionic balance and osmoregulation in plants, but is also closely associated with plant stress resistance [2,3,4]. The concentrations of C, N, P, and K in leaves (the photosynthetic organ) and their ratios largely determine the photosynthesis, growth, reproduction, and ecophysiological processes of plants [3,5,6]. The stoichiometric characteristics of elements in plants can reflect the physiological metabolism and nutrient use efficiency of plants, and their species’ competition mechanisms at the community level [1,2]. They are directly related to the stability and continuity of the ecosystem’s structure and its productivity, and ultimately, affect the energy flow and material cycle of the entire ecosystem [4,7,8,9]. Therefore, it is of great significance to further understand the survival strategies and environmental adaptability of different types of plants [10,11,12], and it is also important for assessing the changes and predictions of biogeochemical cycles and ecosystem functions under global climate change.

The influencing factors of plant stoichiometric characteristics are diverse, including environmental factors such as climate, geography, and soil, as well as biological factors such as plant functional groups, vegetation types, and growth stages. At a large scale, the stoichiometric characteristics of plants change with the variation in mean annual precipitation (MAP), mean annual temperature (MAT), longitude, latitude, and altitude; for instance, at the species or community level, the distribution pattern of leaf N and P concentrations generally decreases with the increase in MAT and MAP [13,14]. Plants and soils are two important components of the biogeochemical cycle, and they are interrelated and interact with each other. Soil properties strongly influence the uptake and utilization of nutrients by plants, and change the stoichiometric characteristics of plant N and P, as well as the overall biomass allocation and ecological strategies of plants [2,5]. Soil nutrients usually have large spatial variability due to various factors (e.g., hydrothermal conditions, vegetation types, succession stages, soil texture, microgeomorphology, etc.) [15,16,17,18], which in turn affect the nutrient traits of plants. With the increase in total soil N:P, the N:P ratios of all plant groups in the grassland of Northern China present a significant downward trend [19]. The concentrations of leaf C, N, and P in plant communities in the desertified area of Northern China are positively correlated with the corresponding soil nutrient contents in the 0–5 cm soil layer, but the correlation strength gradually decreases with the increase in soil depth [20]. For the main conifer species on the eastern margin of the Tibetan Plateau, soil properties may be the main factor influencing leaf C and N:P variation [21]. For desert plants in Xinjiang, most soil factors directly affect N:P in different plant organs, while climatic factors indirectly affect N:P through soil factors [22]. However, there are also many studies that indicate there is no significant relationship between plant leaf nutrient concentrations and soil nutrients [20,22], which reflects the strong internal stability of plant stoichiometry (plants have a strong self-regulation ability in the face of different nutrient conditions) and reflects the influence of their own genetic characteristics [23]. As such, the factors affecting the stoichiometric characteristics of plants at a large scale are complex and comprehensive, and there is no consistent relationship between plants and soil nutrients.

In addition, the plant–soil nutrient relationship is also inevitably related to the research scale, because the smaller the research scale, the simpler the environmental factors, the weaker the interaction, and the easier to quantify the influence of soil factors. By contrast, the effects of soil heterogeneity (corresponding to Stoichiometric Homeostasis Theory) and interspecific phylogeny (corresponding to Species Composition Hypothesis) and individual size (corresponding to Size-Dependent Theory) may be more prominent when climate differences are weakened at small scales [6,13]. For instance, plant stoichiometry exhibits size scaling as the foliar nutrient concentration decreases with increasing plant size, especially for P. Thus, small plants are considered to have lower N:P ratios [24]. For *Quercus acutissima* in three common gardens with different climatic and site conditions, leaf N and P stoichiometry is also significantly correlated with plant size [25]. Therefore, it is necessary to explore the plant–soil relationship in the natural environment with obvious differences in soil texture under the same climatic conditions at the local scale to clearly quantify the influence of differences in soil properties on plant stoichiometric characteristics.

Shrubs with assimilative branches (ABs) are a special group of desert plants whose leaves have degraded or disappeared and photosynthesize mainly with annual ABs, such as species in the genera *Haloxylon*, *Calligonum*, *Ephedra*, *Anabasis*, *Kalidium*, *Halostachys*, and *Halocnemum*. The individual size of these shrubs varies significantly, but the anabolic branches are similar in shape and physiological function, which is the result of a convergence adaptation to the same or similar arid environment. Some species of this shrub are widely distributed in the deserts of Western China, five Central Asian countries, Iran, and even as far as North Africa. They are often the constructive species of desert plant communities and play an important role in sand dune fixation, forage, and ecological security. For instance, *Calligonum mongolicm* and *Haloxylon persicum* are excellent sand-fixing plants in the desert areas of Central Asia, and are also feed for camels, sheep, and many other animals. Reports have shown that although the ABs are formed because of the convergent adaptation of different shrubs to the arid environment, there are obvious interspecific divergent changes in the morphological traits and trait associations [26]. In addition, although the ABs of different shrub species have the same N–P stoichiometric scaling exponent at a large spatial scale, most of the stoichiometric traits vary among species, and their influencing factors are also different [27]. As such, under the same climatic conditions, do the nutrient traits of shrub ABs with different phylogenetic status and individual sizes vary due to soil heterogeneity (corresponding to the stoichiometric homeostasis)? If the variation among sampling sites exists, is the trend consistent for different species (referring to convergence or divergence among species)?

In this study, three sampling sites under the same climatic gradients (mean annual temperature, mean annual precipitation, solar radiation, potential evapotranspiration, aridity index, soil water content, and soil temperature) within 65 km in the southeastern Gurbantunggut Desert in China were selected; the small-sized shrub *Ephedra przewalskii* (EP; Ephedraceae), medium-sized shrub *Calligonum mongolicum* (CM; Polygonaceae), and large-sized shrub *H. persicum* (HP; Amaranthaceae) were chosen; and the plant and soil samples were collected. Our objectives were to (1) compare the differences in stoichiometric traits (nutrient concentrations, stoichiometric ratios, and stoichiometric scaling exponents) in ABs among different sites and species, and to (2) explore the relationship between stoichiometry and soil properties (including nutrients, physical properties, and the biocrust development level) of each species. Based on the existing understanding, we hypothesize that there are obviously interspecific and inter-site (i.e., intraspecific) differences in the nutrient traits in ABs, and there are also interspecific differences in stoichiometric homeostasis and plant–soil relationships, which may be the result of the combined effects of soil heterogeneity (Stoichiometric Homeostasis Theory), plant species (Species Composition Hypothesis), or body size (Size-Dependent Theory). The results can reveal the differences in environmental adaptability and nutrient use strategies of desert shrubs under different soil properties at the local scale, and also provide a scientific basis for the protection and restoration of local desert ecosystems.

## 2. Results

### 2.1. Differences in Soil Properties at Three Sampling Sites

There was no significant difference in very fine particle sizes (*φ* < 0.05 mm and 0.05–0.1 mm) among the three sampling sites, but the contents were all less than 27% (Table 1). In addition, significant differences in the remaining particle sizes among the three sampling sites were determined, and the fine particle size contents of *φ* 0.1–0.25 mm and *φ* < 0.25 mm increased from north to south (i.e., NS < MS < SS), while the coarse particle size contents (*φ* 0.45–1, >1, and >0.45 mm) presented exactly the opposite trend. Therefore, the soil at SS was mainly composed of fine particles (91.38%), whereas the soil coarse particle size content (33.40%) was highest at NS.

In terms of soil nutrients and BSC development, all the parameters presented an increasing trend from north to south, and most values were the lowest at NS and the highest at SS (Table 2). The evaluation of biocrust development indicated that biocrust was at the early-to-intermediate stage of algal crust (DLB = 1.33) at NS, relating to the high content of coarse particle size that was not conducive to the formation of BSC; biocrust was developed from the late algal crust to the early lichen crust (DLB = 3.33) at MS; and biocrust was dominated by lichen crust that was in the intermediate stage (DLB = 5.0) at SS. In general, the soil properties among the three sampling sites varied relatively regularly.

### 2.2. Differences in ABs’ Stoichiometry Among Different Species and Sites

The two-way ANOVA revealed (Table 3) that all 10 nutrient traits showed the highest impact of species; that is, interspecific differences were the most important source of variation in nutrient traits. For most nutrient traits, the sampling site (i.e., habitat heterogeneity) is the secondary influencing factor. In addition, species and site also exhibited significant interactions for all traits.

**Figure 1:**
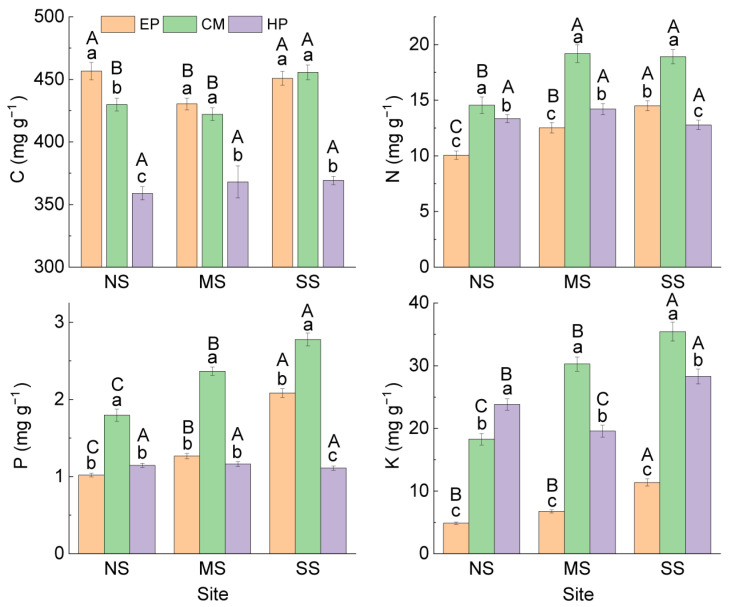
C, N, P, and K concentrations in ABs of three dominant shrubs at three sampling sites in the southeastern Gurbantunggut Desert, China. NS: north site (Cainan); MS: middle site (Guanlichu); SS: south site (Beishawo). EP: *Ephedra przewalskii*; CM: *Calligonum mongolicum*; HP: *Haloxylon persicum*. Different lowercase letters indicate significant (*p* < 0.05) differences between different species at the same site, and different capital letters presented indicate significant (*p* < 0.05) differences between different sites of the same species.

**Figure 2:**
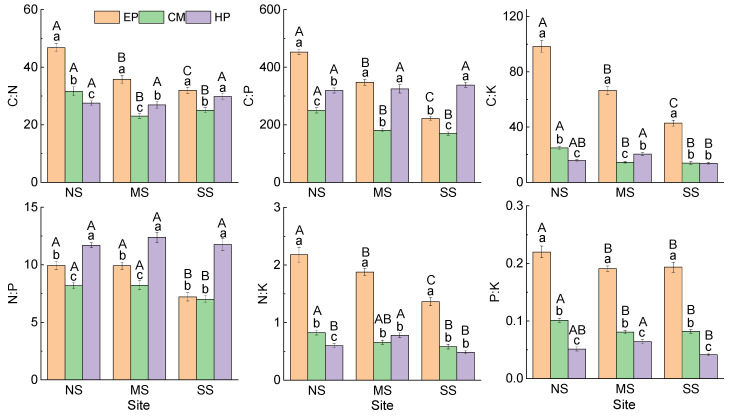
Stoichiometric ratios in ABs of three dominant shrubs at three sampling sites in the southeastern Gurbantunggut Desert, China. NS: north site (Cainan); MS: middle site (Guanlichu); SS: south site (Beishawo). EP: *Ephedra przewalskii*; CM: *Calligonum mongolicum*; HP: *Haloxylon persicum*. Different lowercase letters presented indicate significant (*p* < 0.05) differences between different species at the same site, and different capital letters indicate significant (*p* < 0.05) differences between different sites of the same species.

Comparison of the same species at different sites showed that (Figure 1) there was no significant difference in C concentration in the ABs of EP between NS (456.61 ± 6.91 mg g^−1^) and SS (450.81 ± 5.57 mg g^−1^), and they were significantly higher than that at MS (430.31 ± 4.66 mg g^−1^). The concentrations of N, P, and K in ABs increased significantly from north to south, but all six stoichiometric ratios showed an opposite trend. The nutrient concentrations and stoichiometric ratios in the ABs of CM presented the same trend as that of EP (Figure 2). The stoichiometric characteristics in the ABs of HP at the three sites were different from those of the first two species; there was no significant difference in C, N, or P among the three sites, but K concentration was the highest at SS (28.28 ± 1.20 mg g^−1^) and the lowest at MS (19.56 ± 0.95 mg g^−1^). For stoichiometric ratios of HP, C:K was the lowest at SS; N:K and P:K were the highest at MS. At the same site, there were differences in the change trend of nutrient traits among the three species: C was the lowest in HP; N and P were the highest in CM; K was the lowest in EP; and K of CM was the highest at MS and SS. For stoichiometric ratios, C:N, C:K, N:K, and P:K were all the highest in EP, C:P was the lowest in CM, and N:P was the highest in HP (Figure 2). In summary, there was no consistency in the variation in nutrient traits in ABs among different species and different sites.

**Figure 3:**
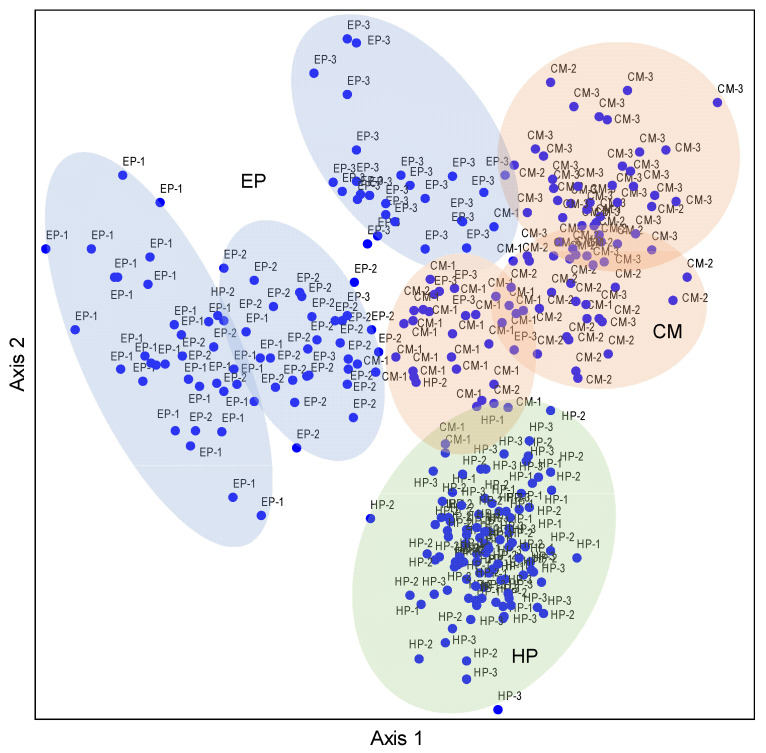
NMDS ordination diagram of three dominant shrubs based on 10 nutrient traits in ABs at three sampling sites in the southeastern Gurbantunggut Desert, China. Number after species: 1, NS, north site (Cainan); 2, MS, middle site (Guanlichu); 3, SS, south site (Beishawo). EP: *Ephedra przewalskii*; CM: *Calligonum mongolicum*; HP: *Haloxylon persicum*. Axis 1 and Axis 2 accounted for 66.7% and 3.8% of the variance, respectively.

The NMDS ordination based on the nutrient matrix indicated that the proportions of variance that accounted for the ordination axes were 66.7% for Axis 1 and 3.8% for Axis 2, accounting for 71.5% of the total variation (Table S2). The three species basically occupied independent space, and they had less overlap but were separated from each other, reflecting obvious interspecific differences. The plant individuals of EP had obvious differentiation among the three sites, especially for NS and SS. The MS site was located between NS and SS, which had a transitional nature (Figure 3). The distribution patterns of CM individuals were similar to those of EP, but the overlap between sampling sites was more than that of EP. The HP individuals were located in the lower part of the NMDS biplot, the individual plants of all three sites were aggregated together, and there was no obvious segregation trend—that is, the overall difference in stoichiometric characteristics of HP plants was small among the three sampling sites. Therefore, the NMDS results intuitively reflected the interspecific differences in the stoichiometric characteristics in ABs of desert shrubs, and also presented the differences or similarities between sampling sites.

### 2.3. Differences in Stoichiometric Scaling Exponents in ABs Among Different Species and Sites

The stoichiometric scaling analysis by sampling site and species (Table S1) showed that only 16/54 trait pairs showed a significant allometric relationship, and most of them were from N vs. P (four pairs) and K vs. P (six pairs). Specifically, the N–C scaling exponent of HP at NS was −2.343, indicating that the C (N) concentration decreased significantly with the increase in N (C) concentration; K and C of HP also showed a similar scaling relationship (slope = −3.943). The P–C scaling exponent of EP at NS was 1.449, indicating that P concentration hyperallometrically increased with the increase in C concentration. Similarly, the K–C of EP at SS (2.684) and N–P of CM at NS (1.361) also showed the same pattern. Moreover, the N–P scaling exponents of EP at MS and SS, and CM at SS were less than 1, showing a hypoallometric pattern between N and P. Isometric relationships were found between K and N of CM at NS and between K and P of EP at MS. Therefore, there were obvious differences in the stoichiometric scaling relationship of different elements between different species at different sites.

The allometric analysis on the pooled data from three sites (Table 4) showed that one-half of the eighteen trait pairs presented a non-significant fitting relationship, e.g., the N–C of all three species, P–C of EP and HP, and K–C of EP and CM. Among the remaining nine significant allometric relationships, seven were hyperallometric, one was isometric, and one was hypoallometric. Specifically, P–C of CM and K-C of HP had the largest allometric growth index (3.411 and 2.814), showing that the allocation rate of P and K was much higher than that of C. In addition, the N–P scaling exponents differed among three shrub species, with EP (slope = 0.712) being hypoallometric, CM being isometric (slope = 1.012), and HP being hyperallometric (slope = 1.281). As such, the pooled data also indicate that obvious differences in the allometric relationships between species and elements exist.

### 2.4. Stoichiometric Homeostasis in the ABs of Three Shrubs

The stoichiometric homeostasis regressions of EP’s C and P:K, CM’s N, N:K, and P:K, and HP’s C, P, C:N, C:P, C:K, and N:P were not significant (*p* > 0.1), so the 1/|*H*| was set to zero (i.e., |*H*| was very large), indicating “strictly homeostatic” (Table 5). The C of CM and N of HP, with |*H*| > 4, were “homeostatic”. The N of EP, C:N and N:P of CM, and K of HP, with 4/3 < |*H*| < 2, were “weakly plastic”. The *|H|* values of the remaining 13 nutrient traits of three species were lower than 4/3, indicating “plastic”. In terms of species, there were two nutrient traits of EP that were “strictly homeostatic”, and the other eight traits were “weakly plastic” and “plastic”; for CM, three traits were “strictly homeostatic”, one was “homeostatic”, and the other six were “weakly plastic” and “plastic”; for HP, six traits were “strictly homeostatic”, one was “homeostatic” and the other three were “plastic”. As such, the overall performance of stoichiometric homeostasis was HP > CM > EP, which showed obvious interspecific differences and was consistent with their body size.

### 2.5. Influencing Factors of ABs’ Stoichiometry of Three Shrubs

**Figure 4:**
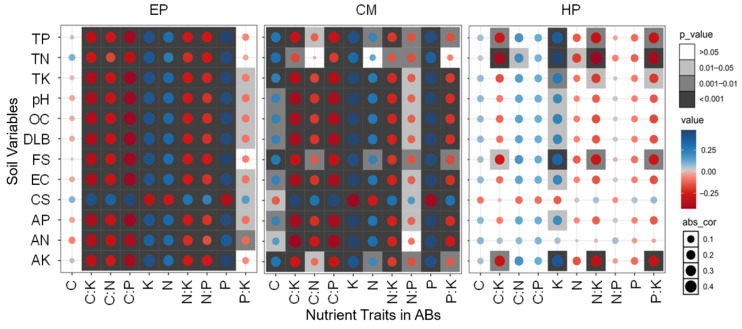
Plots of correlation coefficients between soil variables and nutrient traits in ABs of three dominant shrubs at three sampling sites in the southeastern Gurbantunggut Desert, China. Abs_cor represents the absolute value of the correlation coefficient (0 = no association, 1 = perfect association), quantifying the strength of linear relationships while ignoring directionality, with effect sizes interpreted using Cohen’s benchmark (0.1 = weak, 0.4 = strong). The color gradient from blue to red corresponds to the shift from positive to negative correlations between nutrient traits in ABs and soil variables. EP: *Ephedra przewalskii*; CM: *Calligonum mongolicum*; HP: *Haloxylon persicum*. OC: soil organic carbon content; TN: soil total nitrogen content; TP: soil total phosphorus content; TK: soil total potassium content; AN: soil available nitrogen content; AP: soil available phosphorus content; AK: soil available potassium content; EC: soil electrical conductivity; DLB: developmental level of biocrust; FS: fine-sand content; CS: coarse-sand content.

No significant correlations between the C concentration of EP and any of the soil factors were found; the N, P, and K concentrations were significantly positively correlated with all soil factors (except coarse-sand content); that is, the higher the soil nutrients, biocrust development, and soil fine-sand content, the higher the N, P, and K concentrations of EP (Figure 4). There was a significant negative correlation between coarse-sand content and N, P, and K concentrations of EP, indicating that coarse sand was not conducive to the accumulation of N, P, and K in ABs. Therefore, all five stoichiometric ratios related to C and N showed an opposite trend to soil factors such as N, P, and K. The P:K ratio also had a similar trend (significant correlation with 8/12 factors), but its correlation coefficients were slightly lower. For CM, except for C, the relationship between stoichiometric traits and soil factors was similar to that of EP, but obvious differences in the correlation coefficients exist. Specifically, the correlations between C and soil properties were much stronger, and C, N, P, and K had a basically consistent correlation with the soil factors. Similarly, all six stoichiometric ratios showed an inverse trend with C, N, P, and K. The correlations between the stoichiometric traits of HP and soil factors were relatively weak, and only 26/120 showed significant correlations. On the contrary, C, P, C:P, N:P, and C:N (except C:N and TN) did not show significant correlations with any soil factor. Significant positive correlations between K and 11 soil factors (except CS and AN) were found; that is, the increase in soil nutrients was conducive to K accumulation in the ABs of HP, which could promote its ability to resist environmental stress. In total, the overall association between the nutrient traits of the large-sized shrub HP and soil property was fairly weak, while the associations of the small-sized shrub EP and medium-sized shrub CM were quite strong.

Furthermore, the NMDS result for EP showed that the proportions of variance that accounted for the ordination axes were 74.9% for Axis 1 and 22.3% for Axis 2, accounting for 97.2% of the total variation (Table S2). For CM, the proportions of variance that accounted for the ordination axes were 75.8% for Axis 1 and 3.8% for Axis 2, accounting for 79.6% of the total variation. However, no NMDS result was obtained for HP, indicating that the stoichiometric traits were little affected by surface soil factors (Figure S1). For EP, the distribution patterns of individual plants at three sampling sites in this NMDS biplot were similar to that of the stoichiometric matrix alone (see Figure 3); that is, sites NS, MS, and SS were sequentially distributed from the bottom left to the top right. Soil coarse-sand content was significantly negatively correlated with the sorting axis, and the other 11 soil factors were significantly positively correlated with both axes, indicating that the improvements in soil nutrients and environment contributed to the nutrient enrichment in ABs in EP. By contrast, the distribution of CM individuals was relatively scattered, but the relationship with soil properties was similar to that of EP, except that the influence strength of soil factors (correlation coefficients) was relatively smaller (Table S2). This result also indicated that the nutrients in ABs of EP, which had a smaller body size, were more affected by the soil.

### 2.6. Relative Influences of Different Environmental Types on ABs’ Stoichiometry of Three Shrubs

**Figure 5:**
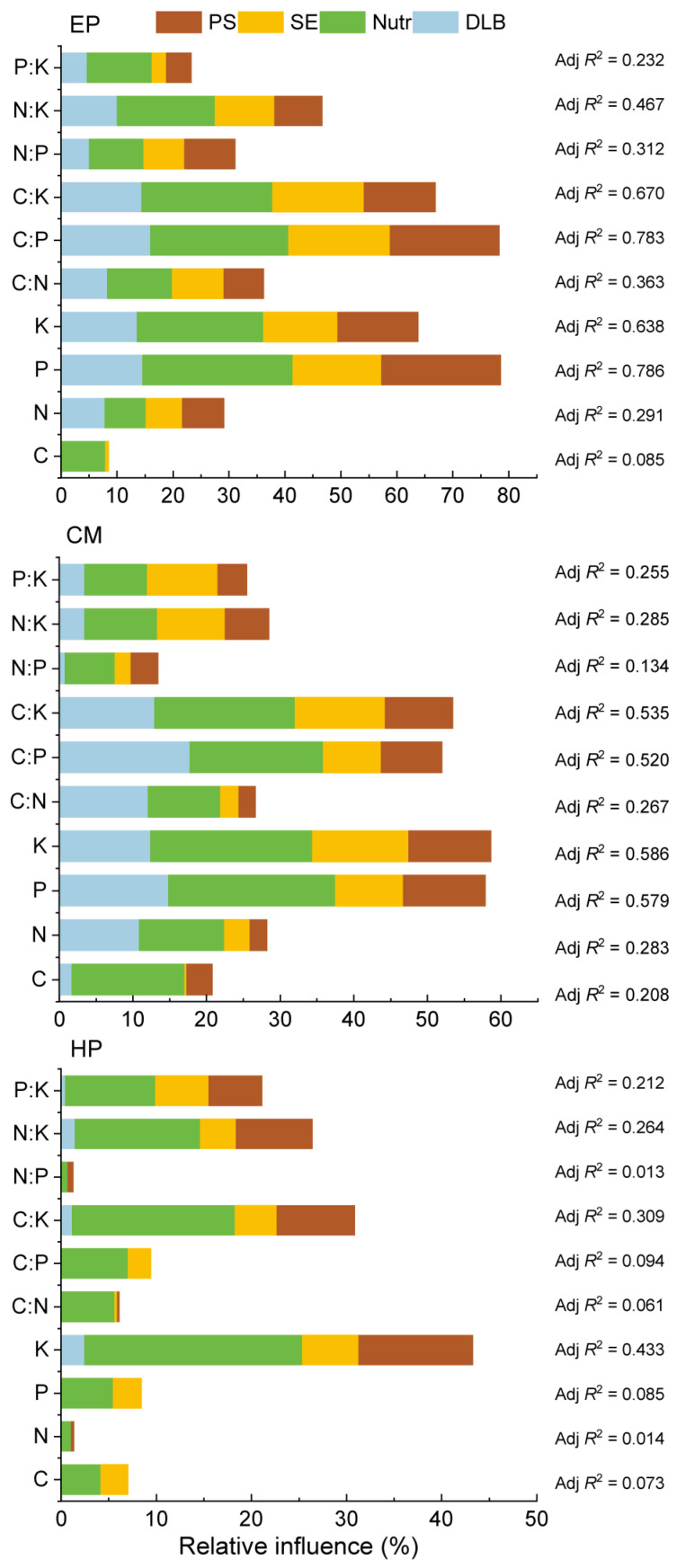
Variance decomposition depicting relative effects (Adj *R*^2^) of soil physical property (PS; soil particle size), soil chemical environment (SE; pH and EC), DLB (developmental level of biocrusts), and soil nutrient variables (nutrients; seven parameters) on 10 nutrient traits in ABs of three dominant shrubs at three sampling sites in the southeastern Gurbantunggut Desert, China. EP: *Ephedra przewalskii*; CM: *Calligonum mongolicum*; HP: *Haloxylon persicum*.

For EP, the P, K, C:P, and C:K accounted for the most variance, all of which exceeded 63%, and both P and C:P exceeded 78% (Figure 5). All four types of soil factors presented a certain relative contribution rate. In addition, N:K and C:N also accounted for more than 30% of the variance, whereas the explanatory powers of other nutrient traits contributed less than 30%. For CM, the P, K, C:P, and C:K accounted for the most variance, all of which exceeded 50%, and the relative contribution rates of soil nutrients and DLB were the largest. The explanatory powers of the other traits were less than 30%. By contrast, for the same nutrient traits (except C and N:P), EP accounted for more variance than CM. For HP, the explanatory powers of only K and C:K exceeded 30%, and the explanatory powers of only N:K and P:K were more than 20% (<30%); among which, soil nutrients showed the highest contribution rate. The explanatory powers of the remaining six traits were all less than 10%, suggesting that soil factors exhibited a very low contribution rate for HP. As such, the explanatory powers of different nutrient traits differed markedly, but overall, EP was the highest, CM was at medium, and HP was the lowest.

## 3. Discussion

### 3.1. Stoichiometry Characteristics in ABs of Three Desert Shrubs Under Different Soil Conditions

Structural elements C and restrictive elements N, P, and K are essential nutrients in the life activities of plant organisms. They play an important role in the process of plant growth, development, and reproduction, and participate in the energy flow and material circulation of the entire ecosystem, thus affecting the succession of the entire terrestrial ecosystem structure [4,6,9,14]. The results of this study showed that there were marked differences in the C, N, P, and K contents and stoichiometric ratios in the ABs of the three desert shrubs, indicating that different desert shrubs had different resource utilization efficiency and adaptation strategies to heterogeneous and unfavorable environments [6].

The 10 nutrient traits in the ABs differed markedly among different species and sampling sites, and the interspecific differences were the most important source of nutrient stoichiometry variation in the three desert shrubs, which is consistent with many reports [28,29,30]. The C contents of *C. mongolicum* and *E. przewalskii* were significantly higher than that of *H. persicum* at the three sites, which was similar to the result of halophytes in an arid desert area [31]. The reason for this may be that salt stress will generally reduce stomatal conductivity [32] and water potential [33], inhibit plant photosynthesis, and thus reduce plant C fixation. Meanwhile, plants need to consume their own heat to cope with salt stress, which leads to a further reduction in plant C content. The contents of N and P of *C. mongolicum* were significantly higher than those of *E. przewalskii* and *H. persicum* at the three sites, which may be due to the clonal growth of *C. mongolicum*, accelerating its growth and metabolic rate. The K element can improve the ability of plants to adapt to adverse environments, and is closely related to the ability of plants to resist drought and disease [34,35,36]. The K content of *E. przewalskii* was significantly lower than that of *C. mongolicum* and *H. persicum*, indicating that the latter two species had stronger resistance than *E. przewalskii*. Evergreen plants have many different leaf properties and nutrient strategies compared to deciduous plants, with deciduous species generally showing higher leaf N and P contents but lower C content than evergreen species [37,38], which is inconsistent with the results of this study. It may be that different species with more distant phylogeny have developed convergent adaptations to some traits due to living in similar arid environmental conditions for a long time. Another reason may be that *E. przewalskii* individuals are the smallest and have a low ability to self-regulate, resulting in a lower K concentration.

The N:P Threshold Hypothesis suggests that there is a specific N:P threshold to determine the nutrient restriction of plant growth. When N:P < 14, plant growth tends to be limited by N; when N:P > 16, plant growth tends to be limited by P; when N:P is between 14 and 16, plant growth may be limited by both N and P, or not by them [39]. N:P < 10 and N:P > 20 are more suitable for determining the nutrient restriction types of N and P by synthesizing more fertilization experimental data. The critical N:P values of the N or P restriction may be different for the different functional groups of plants. Therefore, more and more studies combine the threshold of nutrient stoichiometric ratio, actual content (N, P, K, etc.), soil nutrient status, nutrient scaling exponent, and nutrient reabsorption rate to comprehensively determine the type of plant nutrient restriction. N:K < 2.1 indicates that K is not limited but N is limited; P:K < 0.29 (K:P > 3.4) indicates that K is not limited but P is limited [40]. Based on this standard, all three shrubs had common N and P restrictions, but the degree of N restriction was much higher, which was consistent with the research results of Li et al. [41].

In general, plant nutrient elements are significantly correlated with plant growth [42,43]; however, due to the influence of the external environment, there are significant differences in the absorption, transportation, distribution, and utilization of elements for different species, and the stoichiometric relationships vary with the changes in biotic and abiotic factors [6,44]. In this study, we found significant N–P scaling relationships for the three species at the three sites. The N–P scaling exponents of *E. przewalskii*, *C. mongolicum*, and *H. persicum* at the three sampling sites gradually increased with the individual size of different species, in which the N–P scaling exponent of *E. przewalskii* is was hypoallometric, the N–P scaling exponent of *C. mongolicum* was isometric, and the N–P scaling exponent of *H. persicum* was hyperallometric. The three shrubs did not exhibit the same N–P allometric exponent, which was inconsistent with numerous previous studies. At a large spatial scale for four shrubs with ABs in this desert, a common N–P scaling exponent has been detected, and all N–P scaling exponents are significantly greater than 1 [27]. This may be due to the differences in plant growth rate, leaf property, soil nutrient condition, climate condition, and other factors between the two studies [44,45]. Despite that, all of the N–P allometric exponents of the three shrubs were significantly higher than that of the global mean (2/3 or 3/4) [46,47,48,49], which was consistent with the results in the arid Central Asian desert [27,50]. The power law of N–P scaling suggests that plant resistance is enhanced in high-temperature and drought environments, and the content of resistance-related proteins is increased [6,51,52], i.e., plant adaptability to harsh environments is positively correlated with N input (e.g., allocating more energy for the synthesis of more N-containing stress-resistant compounds), which leads to an increase in the N–P scaling exponent to cope with the environmental stress [7,19,49,50,51,52,53]. Based on the above findings, *H. persicum* was likely more exposed to environmental stress.

Stoichiometric Homeostasis Theory refers to the ability of an organism to maintain a stable concentration and proportion of its own elements in a changing environment [54,55], reflecting the response of biochemical and physiological activities in an organism to the external environment [56]. The stronger stoichiometric homeostasis of plants indicates that plants have a strong ability to control the change in elements, high nutrient utilization efficiency, and can maintain normal growth of the body even in a changeable and barren environment [54], which is also proved by the results of the three shrubs in this study. The order of comprehensive stoichiometric homeostasis of the three species was *H. persicum* > *C. mongolicum* > *E. przewalskii*, which was also consistent with their body size, indicating that the larger species had a stronger ability to regulate the balance and stability of nutrient elements in their bodies. Plants with high stoichiometric homeostasis tend to slow down their own growth rate to maintain normal growth, development, and reproduction [54,57], which may be caused by the difference in plant individual size. In the grassland in Inner Mongolia, one report concludes that species with higher stoichiometric homeostasis have higher dominance and stability, but environmental conditions can change the relationship between stoichiometric homeostasis and ecosystem functions [58]. The *Caragana microphylla* with a higher stoichiometric homeostasis of N has an advantage over *Artemisia halodendron* in the Horqin Sandy Land, indicating that species with high stoichiometric homeostasis are more dominant in competition [59]. Moreover, for herbs, shrubs, and woody plants at the large spatial scale, stoichiometric balance is positively correlated with the stability and function of vegetation [18,60]. As such, for the three shrubs in the present study, *H. persicum* was in a dominant position in the ecological components of the study area, and played a relatively important role in maintaining the stability of the ecosystem.

### 3.2. Soil Properties Differentially Influenced Nutrient Traits in the ABs of Three Desert Shrubs

It is known that significant spatial heterogeneity in soil nutrients in the Junggar Desert exists [56]. This may be related to the extensive coverage of biocrusts (mainly three types: algal, lichen, and moss crusts) with different development degrees and to the differential soil particle sizes in the desert. From north to south in the southeastern part of the desert, the coverage of biological soil crust gradually increases [61,62], and the nutrient contents of the crust-covered soil are significantly higher than those without crusts [17]. However, the soil particle sizes show the opposite trend. In this study, from NS to the MS and then to the SS, soil particle size gradually decreased from the north to the south, biocrust development gradually matured, and soil nutrients also showed an increasing trend; that is, there was a certain correlation between the change in soil nutrient stoichiometry and texture [18,63,64,65]. In the ordination diagram, *E. przewalskii* and *C. mongolicum* individuals at the three sites were both significantly distinguished, confirming that soil heterogeneity markedly influenced the intraspecific differentiation of nutrient use for *E. przewalskii* and *C. mongolicum*, and revealing the interspecific divergence of nutrient use for *H. persicum* and the other two species.

Plant stoichiometric characteristics are closely related to soil physical and chemical properties, climate, plant species, functional groups, study area, and other factors. Most soil factors have direct impacts on plant N and P stoichiometric characteristics, while climate factors indirectly affect plant stoichiometric characteristics mainly by influencing soil factors [22]. In addition, the influences of environmental factors on plant stoichiometry vary with different study scales. At the large spatial scale, multiple environmental factors jointly affect the stoichiometry of herbaceous leaves in the Junggar Desert, in which the influence of climate and geography is greater than that of soil [56]. However, in the Alxa Desert, the explanatory power of soil properties and plant classification for the variation in most leaf element contents is higher than that of climate factors [66]. The leaf stoichiometry of typical desert plants in the northern margin of the Tarim Basin found that soil TN, TP, total dissolved solids, and organic C contents have relatively little influence on leaf stoichiometric characteristics [20,67,68]. That is, at a relatively small scale, the effect of climate factors is weakened and the effect of soil heterogeneity is predictably enhanced. Within a 300 km geographical range, the C, N, and P stoichiometric characteristics of *E. przewalskii*, *C. mongolicum*, and *H. persicum* represent differential variation patterns with longitude and latitude. Soil properties show the greatest influence on *E. przewalskii*, whereas *C. mongolicum* is mainly affected by the interaction of soil properties and climate. Moreover, the climatic, soil, and geographic factors (longitude and latitude) also play different roles in different stoichiometric traits of *H. persicum* [27]. This indicates that different species exhibit different environmental influencing factors, and the changes in plant stoichiometry also tend to reflect more inherent traits of plants [23]. Although this study preliminarily clarified the effects of soil physicochemical properties on the stoichiometric characteristics in the ABs of three desert shrubs under the same climate conditions, the influencing paths and mechanisms need to be further clarified [69,70,71].

From the perspective of the correlation between stoichiometric characteristics in ABs of shrubs and soil, the stoichiometric ratios in ABs of *E. przewalskii* and *C. mongolicum* had very significant correlations with soil. Except that the C of *E. przewalskii* did not change with soil variables, other nutrient traits were mostly affected by soil factors. However, very few significant correlations between stoichiometric traits of *H. persicum* and soil properties were detected, which was consistent with the results of large-scale studies [27]. Combined with the results of regional- and local-scale studies, it is concluded that the structural element C in ABs has strong species-specific characteristics and hardly changes with the changes in external environmental conditions. *H. persicum* belongs to a large shrub (or small tree) with deep roots, and the crown radius of the roots of *H. persicum* can reach 7.1 m. In the vertical direction, *H. persicum* roots can grow to a depth of 5 m (referring to *Haloxylon ammodendron*), and tend to obtain water from deep soil [72,73,74,75]. As such, this study speculated that the plants with a medium or small individual size, such as *E. przewalskii* and *C. mongolicum*, were more affected by soil factors than those with a large individual size, and they also had lower stoichiometric homeostasis (corresponding to Stoichiometric Homeostasis Theory). This supports the view that plants with lower stoichiometric homeostasis are more inclined to adapt to changes in the external environment by adjusting the nutrient content in the body [76,77]. How the plant size influences plant nutrient traits is still not very clear for desert shrubs.

### 3.3. Potential Influence of Plant Size on Nutrient Traits in the ABs of Three Shrubs

Plant size matters to trait associations and links traits to ecosystem multifunctionality, and plant size is also closely related to growth rate [78]. Plant stoichiometry exhibits size scaling, as leaf nutrient concentration decreases with increasing plant size, especially for P [6]. According to the Relative Growth Rate (RGR) theory, if the P content is high and C:P and N:P are low, the RGR is high. Thus, small plants are considered to have lower N:P ratios but a faster growth rate [24]. By contrast, among the three shrubs, *E. przewalskii* exhibited the smallest size, followed by *C. mongolicum*; these both had significantly lower N:P ratios than the large shrub *H. persicum*, which was exactly consistent with the abovementioned hypothesis. However, N, P, and K of *C. mongolicum* were the highest (except K at NS), and C:P and N:P were the lowest, so *C. mongolicum* should have the highest RGR, which may be closely related to the self-regulation mechanism of *C. mongolicum*. Under long-term drought and hot stress, *C. mongolicum* will actively shed some ABs to preserve growth and regeneration potential, and new ABs will sprout again after sufficient rain in late summer and early autumn. In most cases, these re-growing *C. mongolicum* plants can complete fruiting before the next drought. By contrast, *C. mongolicum* and *H. persicum* do not usually have this ability. In the future, in-depth research on the effects of body sizes of shrubs on nutrient traits and the interaction with environmental factors should be carried out, which is crucial to deeply reveal the survival strategies of desert shrubs, especially those with ABs.

The correlation analysis showed that C, C:N (except for SS), and the three K stoichiometric ratios were significantly negatively correlated with individual size, while K (except for pH at the MS site) and N:P were significantly positively correlated with individual size (Figure S2). That is, high K content was closely related to the enhancement of plant resistance, indicating that the large individual plants grow slowly and have weak resistance. For medium- and small-sized plants, especially *C. mongolicum*, they have higher RGR and K-related resistance. This completely confirms the Size-Dependent Theory [24]. Many studies support this view. The concentrations of N, P, and C in the leaves of *Leymus chinensis* and stems significantly increase, negatively correlating with plant size. The N:P of *L. chinensis* is linearly correlated with plant size; however, the ratios of C:N and C:P are positively correlated with plant size, revealing how long-term mowing-induced changes in concentrations, accumulations, ecological stoichiometry, and allocations of key elements are mediated by the variations in plant sizes of perennial rhizome grasses [79]. In addition, the relationship between individual size and stoichiometry also varied among sampling sites in the present study. For instance, in NS, individual size was significantly positively correlated with N content, not significantly correlated in MS, but significantly negatively correlated in SS. The relationship between C:P and individual size was exactly the opposite of that between the N content. Overall, the effects of individual size on plant traits and trait association need to be further focused on.

## 4. Materials and Methods

### 4.1. Study Area

The study area is located in the southeast part (44.470–44.945° N, 87.923–88.393° E) of the Gurbantunggut Desert, which is the second largest desert (4.88 × 10^4^ km^2^) in China, and a typical inland temperate desert in Central Asia. The area is covered with massive, dense, semi-fixed sand dunes approximately 10 m to 50 m high and oriented south to north. The annual precipitation is approximately 70–150 mm, falling predominantly in spring and summer, and snow accumulates 10–30 cm in winter. The mean annual pan evaporation is more than 2000 mm, and the largest amount occurs from April to September. The average annual temperature is 6 °C, with a maximum mean and minimum mean of approximately 30 °C and −20 °C, respectively. The highest wind speed occurs from April to July and is predominantly from the northwest or north direction [80] The soil surfaces in this desert are widely covered by biocrusts. The dune slopes and inter-dune area in the study site are dominated by algal and lichen crusts, respectively; however, the summits of sand dunes are commonly bare sand or have few algal crusts [17,62].

### 4.2. Survey and Collection in ABs of Three Desert Shrubs

**Figure 6:**
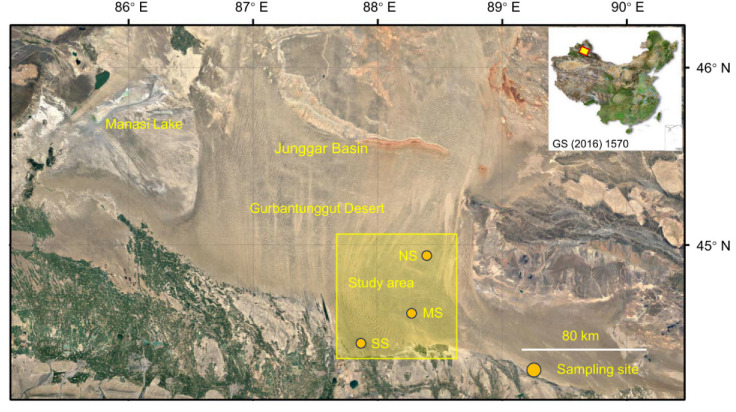
Sampling sites for three dominant shrubs with ABs in the southeastern Gurbantunggut Desert, China. NS: north site (Cainan); MS: middle site (Guanlichu); SS: south site (Beishawo).

At the beginning of September 2021, three study sites, Cainan (denoted as the north site, NS; 44.945° N, 88.393° E), Guanlichu (denoted as the middle site, MS; 44.616° N, 88.266° E), and Beishawo (denoted as the south site, SS; 44.470° N, 87.923° E), were selected, and EP, CM, and HP were well distributed together here. The distance between NS and MS is 38 km, the distance between MS and SS is 31 km, and the distance between NS and SS is 65 km (Figure 6). Based on years of observation, the climatic conditions (including mean annual temperature, mean annual precipitation, solar radiation, potential evapotranspiration, aridity index, soil water content, and soil temperature) of the three sampling sites are basically the same (the detectable rainfall [>0.2 mm] per year was 66.7 mm; the depth of snowfall was approximately 13–15 mm), but there are great differences in the soils’ physical and chemical properties, and differences in soil particle size and biocrust development level are, at most levels, clear [81,82].

**Figure 7:**
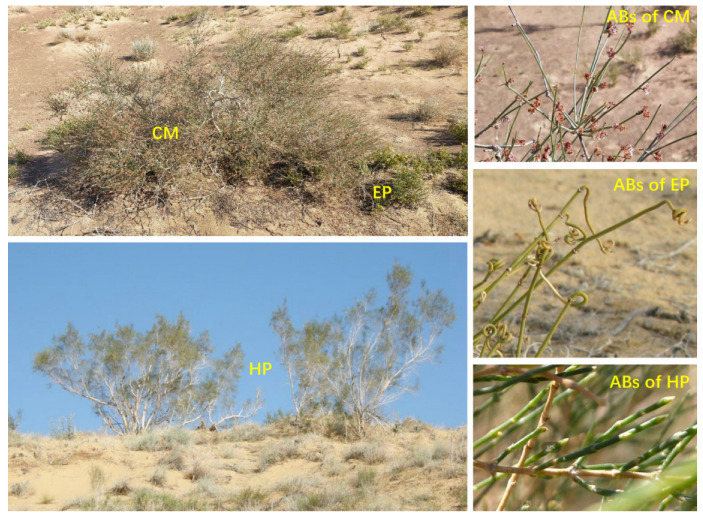
Individuals and ABs of three dominant shrubs in the southeastern Gurbantunggut Desert, China. EP: *Ephedra przewalskii*; CM: *Calligonum mongolicum*; HP: *Haloxylon persicum*.

Six plots with size of 20 m × 20 m were randomly set up for each species at the same site. Five healthy individual plants with similar size were selected for the same species in each plot, and each individual was regarded as one replicate (Figure 7). Due to the uneven distribution of plants, the actual number (replicate) of plants per species at each site varied, with a range of 30 to 38. The total numbers of EP, CM, and HP individuals at the three sites were 92, 95, and 104, respectively. Subsequently, the fully matured and sun-exposed ABs in the middle canopy were collected. Plant samples were then placed inside a foam box containing ice bags, and transported immediately to the laboratory. Plant size was also measured during the field survey. The mean crown diameters of EP, CM, and HP were 18.61 ± 1.12, 94.68 ± 10.12, and 185.41 ± 15.45 cm, respectively; and the average plant heights were 16.82 ± 0.95, 57.20 ± 4.43, and 223.47 ± 19.38 cm, respectively.

### 4.3. Determination of C, N, P, and K Concentrations in ABs

Plant samples were dried in an oven at 75 °C to a constant weight. The dried assimilative branch samples were milled into powder in a vibratory disc mill (MM400, Retsch GmbH Inc., Haan, Germany) and stored in zip bags. The leaf C (mg g^−1^) and N (mg g^−1^) concentrations were measured using an elemental analyzer (Multi N/C 3100, Analytik Jena AG, Jena, Germany). Leaf P (mg g^−1^) concentration was measured using the molybdenum–antimony anti-spectrophotometric method. Leaf K (mg g^−1^) concentration was measured by flame spectrophotometry (FP640, Jingke Co., Shanghai, China). The stoichiometric ratios were then calculated.

### 4.4. Collection and Determination of Soils

In order to compare the differences in soil properties between the three sites, soil samples were collected, and the biocrust development was evaluated. One mixed soil sample (five-point sampling method) at a depth of 0–10 cm was collected in each 20 m × 20 m plot. Soils were air-dried in the laboratory and then sieved. First, the soil particle size was determined by sieving method, and the sieve hole sizes were 0.05 mm, 0.1 mm, 0.25 mm, 0.45 mm, and 1 mm. In order to explore the relationship between soil particle size and plant nutrients more clearly, the particle size *φ* < 0.25 mm was defined as fine sand, and *φ* > 0.45 mm was regarded as coarse sand [83,84]. Next, the concentrations of soil organic carbon (OC, g kg^−1^), total nitrogen (TN, g kg^−1^), total phosphorus (TP, g kg^−1^), total potassium (TK, g kg^−1^), available N (AN, mg kg^−1^), available P (AP, mg kg^−1^), available K (AK, mg kg^−1^), soil pH (water–soil ratio of 1:2.5), and electrical conductivity (EC, μS cm^−1^; water–soil ratio of 1:5) were determined in accordance with standard methods referenced from Bao (2000) [85].

Moreover, the developmental levels of biocrusts are a crucial indicator to reflect soil nutrient status and the plant–soil relationship in this desert [86]; thus, biocrusts are considered as an important soil biological factor in the present study. Algal and moss crusts are at early and late developmental stages of biocrusts, respectively, and intermixed with many intermediate stages [87]. On the basis of Belnap et al. [87], we further developed the evaluation system of the development level of biocrust (DLB) based on their type, color, and roughness using a non-destructive approach. Here, bare sand was recorded as DLB = 0 and pure moss crust as DLB = 9, with eight other intermediate stages between bare sand and crust [17,88]. The higher the DLB, the greater the development levels of biocrusts and the higher the soil nutrient contents [17].

### 4.5. Calculation of Allometric Growth and the Stoichiometric Homeostasis Index

The allometric model (power function: *Y* = *βXα*) features *Y*/*X* as traits, *β* as the constant, and α as an allometric exponent. Two potential outcomes emerged: (1) an isometric relationship between *Y* and *X* (where *α* showed no significant difference from 1); and (2) an allometric relationship (with a hyperallometric relationship when α was significantly < 1, and a hypoallometric relationship when *α* was significantly > 1). Computational procedures involved log-transforming both sides of the equation to log*Y* = log*β* + *α*log*X*, followed by standardized major axis estimation (SMA) using the “smart” package in R v4.2.2. This generated the regression coefficient of determination (*R*^2^), 95% confidence intervals (95% *CI*) for *α*, allometric index difference tests, and isometry tests, with only significantly fitted allometric equations retained for further analysis.

An organism’s degree of stoichiometric homeostasis was characterized by the homeostasis coefficient *H*: *y* = *cx*1/*H*, where *x* is the resource (i.e., soil) nutrient variable (e.g., TN, TP, or TK), *y* is the organism’s nutrient variable (same units as resource), and *c* is a constant [4]. Through field experiments, it was determined that the stoichiometric homeostasis index of organisms may have a negative value [77], so the absolute value |*H*| of the stoichiometric homeostasis index *H* is required to characterize the strength of stoichiometric homeostasis in organisms. Therefore, 1/|*H*| is the slope of the regression between log(*x*) and log(*y*). Since the slope was expected to be greater than or equal to 0, one-tailed tests with *α* = 0.1 were used. If the regression relationship was non-significant (*p* > 0.1), 1/|*H*| was set to zero (indicating that |*H*| was very large) and the organism considered “strictly homeostatic”. Species with 1/|*H*| = 1 were not homeostatic. All datasets with significant regressions and 0 < 1/|*H*| < 1 were arbitrarily classified as |*H*| > 4 “homeostatic”; 2 < |*H*| < 4 “weakly homeostatic”; 4/3 < |*H*| < 2 “weakly plastic”; |*H*| < 4/3 “plastic” [77]. The homeostasis index was calculated using R 4.2.2.

### 4.6. Statistical Analysis

The normal distribution of each parameter was determined by the one-sample Kolmogorov–Smirnov test. Two-way ANOVA was used to assess the primary and interactive effects of species versus sites. The differences in each stoichiometric trait between three sites and between three species were compared using one-way ANOVA (*α* = 0.05). Levene’s test was used to test the homogeneity, and post hoc multiple comparisons were performed using Duncan’s test. Pearson’s correlation analysis was used to determine the relationship between soil properties and stoichiometric traits in ABs using R 4.2.2.

Non-metric multidimensional scaling (NMDS) ordination was employed to comprehensively explore the differentiation of the three shrubs at the three sampling sites based on a stoichiometric matrix using PC-ORD version 7.08 (MjM Software Design, Gleneden Beach, Oregon). Moreover, the NMDS ordination was also used to distinguish the overall relationship between the stoichiometric trait matrix and soil property matrix for each species at the three sampling sites. To quantify the variance explanatory powers of nutrient traits in the ABs of each species, each nutrient trait was used as the response variable, and the environmental factors (soil physical property [soil particle size], soil chemical environment [pH and EC], DLB [a biotic factor], and soil nutrient [seven parameters]) were used as the explanatory variables. The variance decomposition was run to determine the relative effects of four groups of explanatory variables on plant nutrient traits using the “rdacca.hp” package in R 4.2.2.

The SPSS 26.0 statistical package (SPSS Inc., Chicago, IL, USA) was used for common data analyses. Graphs were made using Origin Pro 2021.

## 5. Conclusions

The present study evaluated the differences in ABs’ stoichiometry of three different desert shrubs under heterogeneous soil conditions at a small scale in a temperate desert in Central Asia. The stoichiometric characteristics of ABs were most affected by species, followed by soil heterogeneity, and a significant interaction between the two factors also existed. The small-sized shrub *E. przewalskii* had high C, C:N, C:P, C:K, N:K, and P:K, but low N, P, K, and N:P; however, the large-sized shrub *H. persicum* had the highest N:P, which conformed to the Size-Dependent Theory and Growth Rate Hypothesis. The C of *E. przewalskii*, C and N of *C. mongolicum*, and C, N, and P of *H. persicum* in the ABs of three species showed high stoichiometric homeostasis, and the order of comprehensive stoichiometric homeostasis was *H. persicum* > *C. mongolicum* > *E. przewalskii*. The N–P scaling exponent of *H. persicum* was significantly higher than 1 (exactly corresponding to the highest N:P), and it had high K concentration, which corresponded to high resistance but low growth rate. *E. przewalskii* and *C. mongolicum* individuals were significantly separated among the three sites in the ordination diagram (*H. persicum* was aggregated), most nutrient traits of these plants were closely related to soil factors, and the environmental interpretation percentages were generally higher than *H. persicum*. In addition, the individual size and rooting depth were likely an important source of the variation in the ABs’ stoichiometry for different species. The results of this study confirm the existence of intraspecific and interspecific differences in ABs’ stoichiometry of different desert shrubs under heterogeneous soil conditions at a small scale, which is of great significance for further understanding the convergent or divergent adaptation and survival strategies of desert shrubs.

## Figures and Tables

**Table 1:** Proportion (%) of soil particle size content at three sampling sites in the southeastern Gurbantunggut Desert, China.

Site	<0.05 mm	0.05–0.1 mm	0.1–0.25 mm	0.25–0.45 mm	0.45–1 mm	>1 mm	>0.45 mm	<0.25 mm
NS	1.40 ± 0.16 a	21.56 ± 2.45 a	28.78 ± 2.45 c	14.29 ± 1.29 b	32.43 ± 2.47 a	0.97 ± 0.29	33.40 ± 2.64 a	51.73 ± 2.68 c
MS	2.09 ± 0.13 a	20.35 ± 1.20 a	40.36 ± 2.46 b	25.97 ± 2.30 a	10.24 ± 1.17 b	0	10.24 ± 1.17 b	62.79 ± 3.32 b
SS	2.19 ± 0.56 a	26.98 ± 5.45 a	62.21 ± 4.67 a	7.71 ± 2.10 c	0.156 ± 0.04 c	0	0.16 ± 0.04 c	91.38 ± 2.03 a

NS: north site (Cainan); MS: middle site (Guanlichu); SS: south site (Beishawo). Different lowercase letters in the same column indicate significant differences (*p* < 0.05).

**Table 2:** Soil nutrients and developmental levels of biocrust (DLB) at three sampling sites in the southeastern Gurbantunggut Desert, China.

Index	Site		
	NS	MS	SS
OC (g kg^−1^)	0.73 ± 0.06 b	1.03 ± 0.04 ab	1.32 ± 0.19 a
TN (g kg^−1^)	0.12 ± 0.01 b	0.11 ± 0.01 b	0.17 ± 0.01 a
TP (g kg^−1^)	0.28 ± 0.02 b	0.31 ± 0.03 b	0.39 ± 0.02 a
TK (g kg^−1^)	10.46 ± 0.14 c	11.78 ± 0.15 b	13.39 ± 0.18 a
AN (mg kg^−1^)	0.33 ± 0.03 b	0.48 ± 0.06 a	0.51 ± 0.03 a
AP (mg kg^−1^)	1.91 ± 0.18 b	2.98 ± 0.18 a	3.76 ± 0.41 a
AK (mg kg^−1^)	83.0 ± 4.07 b	94.17 ± 12.56 b	150.0 ± 7.70 a
pH	7.76 ± 0.07 c	8.10 ± 0.05 b	8.36 ± 0.03 a
EC (μS cm^−1^)	44.83 ± 1.96 b	60.17 ± 6.05 a	73.00 ± 5.50 a
DLB	1.33 ± 0.33 c	3.33 ± 0.33 b	5.0 ± 0.36 a

NS: north site (Cainan); MS: middle site (Guanlichu); SS: south site (Beishawo). OC: soil organic carbon content; TN: soil total nitrogen content; TP: soil total phosphorus content; TK: soil total potassium content; AN: soil available nitrogen content; AP: soil available phosphorus content; AK: soil available potassium content; pH: soil potential of hydrogen; EC: soil electrical conductivity; DLB: developmental level of biocrust. Different lowercase letters in the same row indicate significant differences (*p* < 0.05).

**Table 3:** Interaction of species and site on AB stoichiometry of three dominant shrubs at three sampling sites in the southeastern Gurbantunggut Desert, China. F, measures the ratio of between-group variance to within-group variance. Larger values indicate stronger factor effects; P, probability of observing the result under the null hypothesis. *p* < 0.05 is considered statistically significant.

Source	C		N		P		K		C:N	
	*F*	*P*	*F*	*P*	*F*	*P*	*F*	*P*	*F*	*P*
Site	5.14	0.006	24.01	2.35 × 10^−10^	127.91	2.92e × 10^−40^	72.25	4.65 × 10^−26^	31.60	4.13 × 10^−13^
Species	119.65	2.38 × 10^−38^	73.01	2.82 × 10^−26^	421.13	2.06 × 10^−85^	355.60	7.99 × 10^−78^	86.03	6.78 × 10^−30^
Site ∗ Species	2.85	0.0241	8.16	3.07 × 10^−6^	40.08	1.42 × 10^−26^	21.15	2.87 × 10^−15^	14.46	9.38 × 10^−11^
Source	C:P		C:K		N:P		N:K		P:K	
	*F*	*P*	*F*	*P*	*F*	*P*	*F*	*P*	*F*	*P*
Site	82.05	8.21 × 10^−29^	112.20	1.42 × 10^−36^	14.74	8.16 × 10^−7^	34.81	3.07 × 10^−14^	9.56	9.67 × 10^−5^
Species	205.59	8.43 × 10^−56^	758.61	3.3 × 10^−114^	103.46	2.01 × 10^−34^	361.05	1.71 × 10^−78^	646.09	5.02 × 10^−106^
Site ∗ Species	46.81	3.78 × 10^−30^	62.01	1.53 × 10^−37^	4.05	0.0033	11.61	9.62e × 10^−9^	4.80	0.001

**Table 4:** Allometric scaling slopes of C, N, P, and K (*y* vs. *x*) in ABs among three dominant shrubs across three sampling sites in the southeastern Gurbantunggut Desert, China.

Elemental Pair	Species	*R* ^2^	*p*	Slope	S.D.	Type
N–C	EP	0.008	0.393	–	–	–
	CM	0.007	0.405	–	–	–
	HP	0.003	0.572	–	–	–
P–C	EP	0.006	0.472	–	–	–
	CM	0.069	0.010	3.411	0.678	Hyperallometric
	HP	0.000	0.935	–	–	–
K–C	EP	0.008	0.406	–	–	–
	CM	0.022	0.152	–	–	–
	HP	0.053	0.019	2.814	1.174	Hyperallometric
N–P	EP	0.382	0.000	0.712 b	0.117	Hypoallometric
	CM	0.292	0.000	1.012 a	0.175	Isometric
	HP	0.119	0.000	1.281 a	0.236	Hyperallometric
K–N	EP	0.390	0.000	1.767 a	0.289	Hyperallometric
	CM	0.201	0.000	1.443 a	0.266	Hyperallometric
	HP	0.004	0.544	–	–	–
K–P	EP	0.712	0.000	1.258 a	0.142	Hyperallometric
	CM	0.646	0.000	1.460 a	0.179	Hyperallometric
	HP	0.016	0.207	–	–	–

EP: *Ephedra przewalskii*; CM: *Calligonum mongolicum*; HP: *Haloxylon persicum*. Different lowercase letters after scaling slopes indicate significant (*p* < 0.05) differences in a nutrient pair among different shrubs. “–” indicates insignificant allometric scaling relationships.

**Table 5:** The stoichiometric homeostatic index (|*H*|) in ABs of three dominant shrubs across three sampling sites in the southeastern Gurbantunggut Desert, China.

Species	|*H*_C_|	|*H*_N_|	|*H*_P_|	|*H*_K_|	|*H*_C:N_|	|*H*_C:P_|	|*H*_C:K_|	|*H*_N:P_|	|*H*_N:K_|	|*H*_P:K_|
EP	–	1.894	0.445	0.294	1.221	0.907	0.420	0.229	1.093	–
CM	10.870	–	0.824	0.384	1.425	0.767	0.552	1.736	–	–
HP	–	5.236	–	1.484	–	–	–	–	0.706	0.243

EP: *Ephedra przewalskii*; CM: *Calligonum mongolicum*; HP: *Haloxylon persicum*. “–” indicates 1/|*H*| was set to zero (i.e., |*H*| was very large); thus, the stoichiometric trait was “strictly homeostatic”. All datasets with significant regressions and 0 < 1/|*H*| < 1 were arbitrarily classified as |*H*| > 4 “homeostatic”; 2 < |*H*| < 4 “weakly homeostatic”; 4/3 < |*H*| < 2 “weakly plastic”; |*H*| < 4/3 “plastic”.

## Data Availability

The data that support the findings of this study are available from the corresponding authors upon reasonable request.

## Supplementary Materials

The following supporting information can be downloaded at https://www.mdpi.com/article/10.3390/plants14213363/s1. Table S1: Allometric scaling slopes among C, N, P, and K of assimilative branches of three shrubs in three sampling sites in the southeastern Gurbantunggut Desert, China; Table S2: Correlation coefficients between nutrient traits of assimilative branches of three shrubs and the first two axes of NMDS ordination across three sampling sites in the southeastern Gurbantunggut Desert, China; Table S3 Correlation coefficients between nutrient traits of assimilative branches of *Ephedra przewalskii* and *Calligonum mongolicum* and soil variables and the first two axes of NMDS ordination in three sampling sites in the southeastern Gurbantunggut Desert, China; Figure S1: NMDS ordination diagrams between soil property matrix and stoichiometric trait matrix of *Ephedra przewalskii* (EP) and *Calligonum mongolicum* (CM) in three sampling sites in the southeastern Gurbantunggut Desert, China. The sampling individuals and soil variables were shown in the diagrams. 1–NS: north site (Cainan); 2–MS: middle site (Guanlichu); 3–SS: south site (Beishawo). OC: soil organic carbon content; TN: soil total nitrogen content; TP: soil total phosphorous content; TK: soil total potassium content; AN: soil available nitrogen content; AP: soil available phosphorous content; AK: soil available potassium content; EC: soil electrical conductivity; IBL: developmental level of biocrust; Figure S2: Correlation coefficients between stoichiometric traits of assimilative branches and plant size of three shrubs in three sampling sites in the southeastern Gurbantunggut Desert, China. NS: north site (Cainan); MS: middle site (Guanlichu); SS: south site (Beishawo).
