# Supplementary material for: Local-Scale Soil Heterogeneity Differentially Influenced Assimilative Branch Stoichiometry of Three Dominant Shrubs in a Central Asian Desert
Source: Plants (Basel). 2025 Nov 3;14(21):3363. doi: 10.3390/plants14213363 (PMC12610744; doi:10.3390/plants14213363)
Supplement: Supplementary file 1 [file plants-14-03363-s001.zip › plants-3867408-supplementary.pdf]

Supplementary material

Local-scale soil heterogeneity differentially influenced assimilative branch stoichiometry of three dominant shrubs in a central Asian desert

Plant and soil

Cheng-Cheng Wang [1,2,3], Xue-Lian Zhang [2,3], Ye Tao [2,3,*], Ling Dai [2,3,4], Huan-Huan Meng [2,3], Xiao-Bing Zhou [2,3] and Yuan-Ming Zhang [2,3,*]

[1] College of Life Sciences, Tarim University, Alar 843300, China; wangchengcheng@163.com
[2] State Key Laboratory of Ecological Safety and Sustainable Development in Arid Lands, Xinjiang Institute of Ecology and Geography, Chinese Academy of Sciences, Urumqi 830011, China; zhangxuelian@163.com (X.-L.Z.); dailing_2025@163.com (L.D.); menghuanhuan1214@163.com (H.-H.M.); zhouxb@ms.xjb.ac.cn (X.-B.Z.)
[3] Xinjiang Key Laboratory of Biodiversity Conservation and Application in Arid Lands, Xinjiang Institute of Ecology and Geography, Chinese Academy of Sciences, Urumqi 830011, China
[4] Xinjiang Key Laboratory of Special Species Conservation and Regulatory Biology, Key Laboratory of Special Environment Biodiversity Application and Regulation in Xinjiang, Key Laboratory of Plant Stress Biology in Arid Land, College of Life Sciences, Xinjiang Normal University, Urumqi 830054, China
* Correspondence: taoye@ms.xjb.ac.cn (Y.T.); ymzhang@ms.xjb.ac.cn (Y.-M.Z.)

Table S1 Allometric scaling slopes among C, N, P, and K of assimilative branches of three shrubs in three sampling sites in the southeastern Gurbantunggut Desert, China

Elemental pair	Site	Species	R^2	P	Slope	S.D.
N-C	NS	EP	0.020	0.459	–	–
		CM	0.010	0.600	–	–
		HP	0.132	0.041	−2.343	0.814
	MS	EP	0.001	0.890	–	–
		CM	0.000	0.913	–	–
		HP	0.005	0.681	–	–
	SS	EP	0.005	0.694	–	–
		CM	0.008	0.597	–	–
		HP	0.001	0.901	–	–
P-C	NS	EP	0.191	0.018	1.449	0.515
		CM	0.000	0.947	–	–
		HP	0.000	0.915	–	–
	MS	EP	0.103	0.084	–	–
		CM	0.002	0.812	–	–
		HP	0.000	0.965	–	–
	SS	EP	0.007	0.630	–	–
		CM	0.000	0.904	–	–
		HP	0.069	0.152	–	–
K-C	NS	EP	0.001	0.844	–	–
		CM	0.000	0.963	–	–
		HP	0.186	0.014	−3.943	1.326
	MS	EP	0.012	0.567	–	–
		CM	0.021	0.442	–	–
		HP	0.003	0.744	–	–
	SS	EP	0.183	0.010	2.684	0.859
		CM	0.035	0.264	–	–
		HP	0.047	0.240	–	–
N-P	NS	EP	0.103	0.089	–	–
		CM	0.382	0.000	1.361	0.414
		HP	0.111	0.063	–	–
	MS	EP	0.417	0.000	0.822	0.243
		CM	0.001	0.886	–	–
		HP	0.016	0.474	–	–
	SS	EP	0.503	0.000	0.847	0.211
		CM	0.142	0.020	0.794	0.249
		HP	0.008	0.639	–	–
K-N	NS	EP	0.000	0.944	–	–
		CM	0.444	0.000	1.024	0.296
		HP	0.015	0.508	–	–
	MS	EP	0.129	0.051	–	–
		CM	0.008	0.636	–	–
		HP	0.001	0.859	–	–

	SS	EP	0.015	0.478	–	–
		CM	0.114	0.038	1.541	0.491
		HP	0.049	0.234	–	–
K-P	NS	EP	0.000	0.938	–	–
		CM	0.617	0.000	1.393	0.334
		HP	0.169	0.019	1.674	0.570
	MS	EP	0.328	0.001	1.123	0.357
		CM	0.175	0.021	1.583	0.557
		HP	0.461	0.000	1.589	0.414
	SS	EP	0.009	0.580	–	–
		CM	0.188	0.007	1.942	0.592
		HP	0.027	0.376	–	–

NS: north site (Cainan); MS: middle site (Guanlichu); SS: south site (Beishawo).

Table S2 Correlation coefficients between nutrient traits of assimilative branches of three shrubs and the first two axes of NMDS ordination across three sampling sites in the southeastern Gurbantunggut Desert, China

Trait	Axis 1	Axis 2
C	−0.309***	0.801***
N	0.626***	0.382***
P	0.711***	0.802***
K	0.685***	0.088
C:N	−0.776***	0.057
C:P	−0.938***	−0.502***
C:K	−0.770***	0.142*
N:P	−0.309***	−0.702***
N:K	−0.609***	0.144*
P:K	−0.479***	0.414***
Variation explained	67.7%	3.8%

*: $P < 0.05$; **: $P < 0.01$; ***: $P < 0.001$.

Table S3 Correlation coefficients between nutrient traits of assimilative branches of *Ephedra przewalskii* and *Calligonum mongolicum* and soil variables and the first two axes of NMDS ordination in three sampling sites in the southeastern Gurbantunggut Desert, China

Parameter	*Ephedra przewalskii* (EP)		*Calligonum mongolicum* (CM)	
	Axis 1	Axis 2	Axis 1	Axis 2
Stoichiometric trait				
C	0.086	−0.580***	−0.105	0.958***
N	0.590***	0.649***	0.527***	0.196
P	0.972***	0.714***	0.894***	0.479***
K	0.846***	0.762***	0.806***	0.352***
C:N	−0.589***	−0.796***	−0.643***	0.041
C:P	−0.953***	−0.881***	−0.981***	−0.252*
C:K	−0.809***	−0.829***	−0.841***	−0.217*
N:P	−0.632***	−0.298**	−0.288***	−0.300**
N:K	−0.619***	−0.444***	−0.428***	−0.267**
P:K	−0.163	−0.337**	−0.337**	−0.081
Soil variable				
DLB	0.884***	0.756***	0.634***	0.464***
OC	0.891***	0.753***	0.625***	0.472***
TN	0.809***	0.521***	0.326**	0.503***
TP	0.903***	0.683***	0.512***	0.514***
TK	0.902***	0.743***	0.603***	0.486***
AN	0.778***	0.746***	0.682***	0.374***
AP	0.876***	0.759***	0.643***	0.456***
AK	0.894***	0.659***	0.482***	0.517***
pH	0.879***	0.758***	0.640***	0.459***
EC	0.885***	0.756***	0.634***	0.465***
Fine sand	0.907***	0.698***	0.533***	0.510***
Coarse sand	−0.836***	−0.760***	−0.668***	−0.420***
Variation explained	74.9%	22.3%	75.8%	3.8%

*: $P < 0.05$; **: $P < 0.01$; ***: $P < 0.001$.

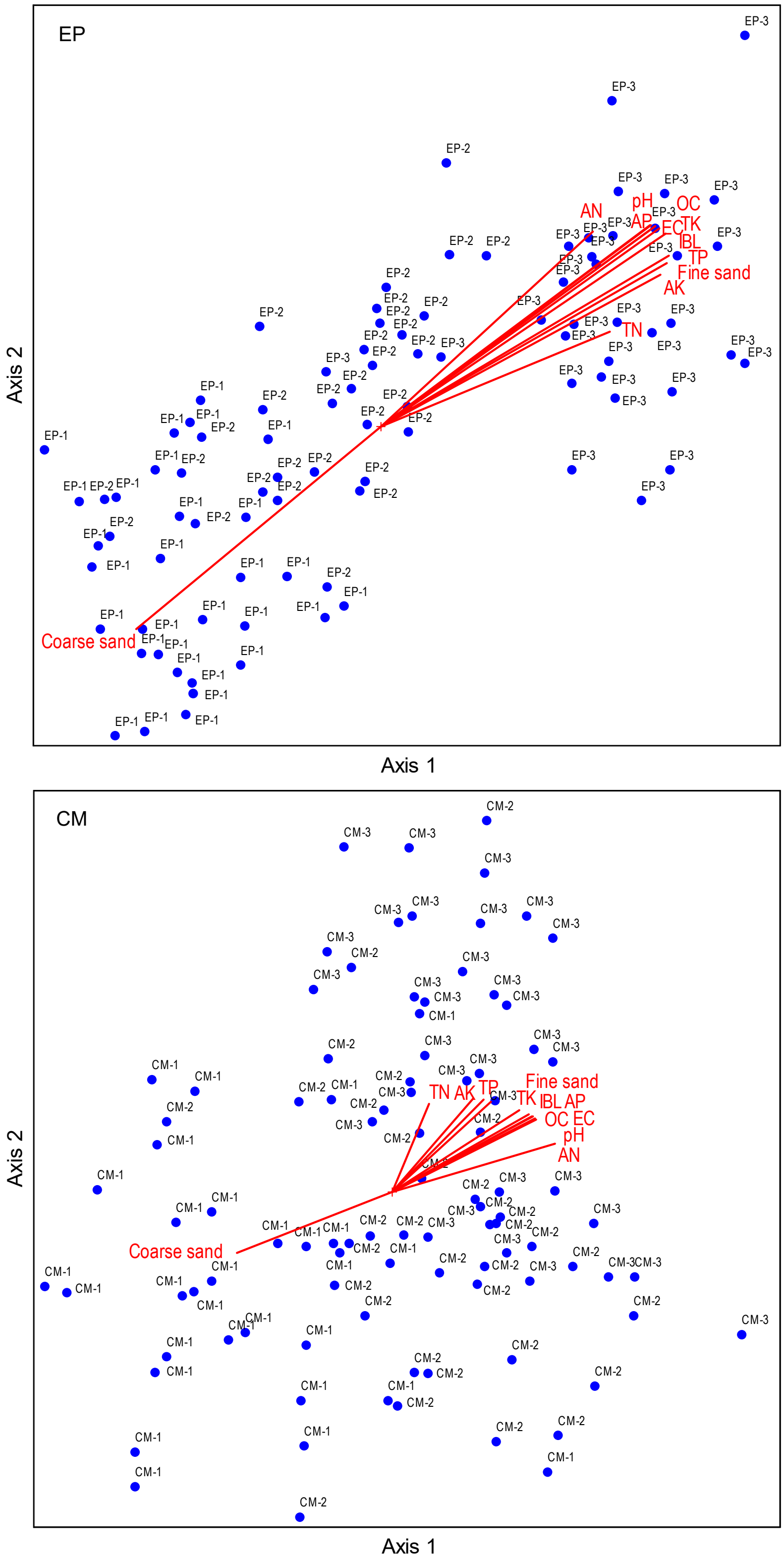

Figure S1 NMDS ordination diagrams between soil property matrix and stoichiometric trait matrix of *Ephedra przewalskii* (EP) and *Calligonum mongolicum* (CM) in three sampling sites in the southeastern Gurbantunggut Desert, China. The sampling individuals and soil variables were shown in the diagrams. 1–NS: north site (Cainan); 2–MS: middle site (Guanlichu); 3–SS: south site (Beishawo). OC: soil organic carbon content; TN: soil total nitrogen content; TP: soil total phosphorous content; TK: soil total potassium content; AN: soil available nitrogen content; AP: soil available phosphorous content; AK: soil available potassium content; EC: soil electrical conductivity; IBL: developmental level of biocrust.

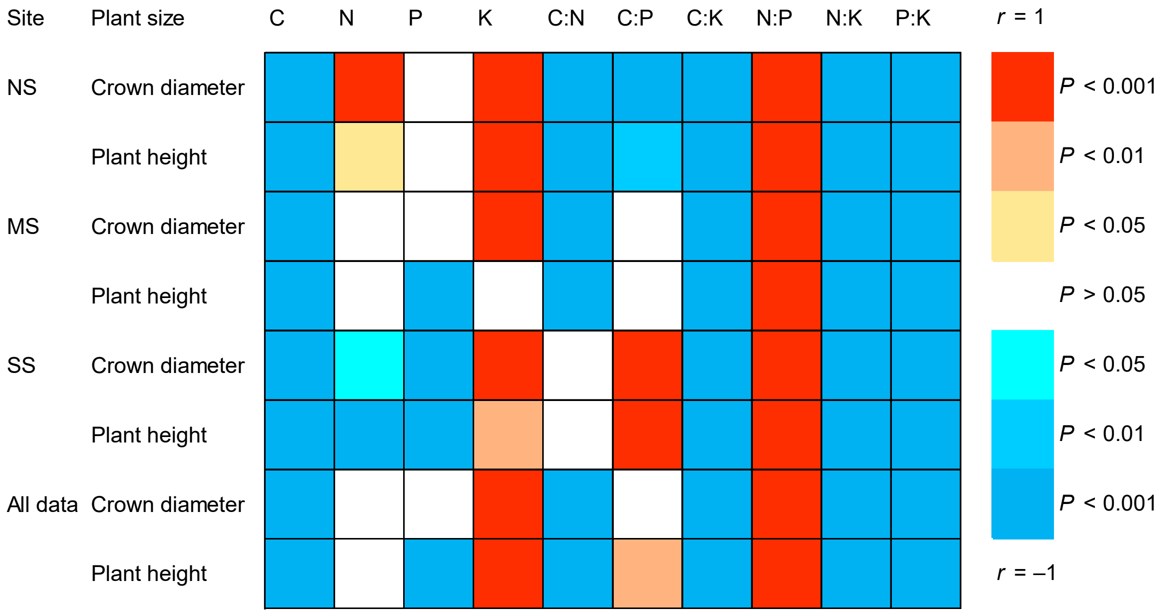

Figure S2 Correlation coefficients between stoichiometric traits of assimilative branches and plant size of three shrubs in three sampling sites in the southeastern Gurbantunggut Desert, China. NS: north site (Cainan); MS: middle site (Guanlichu); SS: south site (Beishawo).
